# Medical Miscellany

**Published:** 1919-06

**Authors:** 


### MEDICAL MISCELLANY

### Medical Men Discuss Adoption of R. O. T. C.

Dr. E. H. Cary, dean of Baylor Medical College, has returned from Chicago, where he has been attending the National Association of American Medical Colleges. One of the principal plans discussed for medical colleges of the United States was the probable adoption of the Reserve Officers' Training Corps.

"This plan," said Dr. Carey, "was offered by the educational section of the War Department for discussion in medical colleges. The Reserve Officers Training Corps may be established in class A medical schools, if the colleges desire. The plan has not yet been adopted by the general staff of the army, but if it proves acceptable Baylor will seriously discuss the advantages of becoming one of the Reserve Officers Training Corps schools.

"The college must have as many as 100 students in order to consider the proposition. The advantages of the plan will lie in the stipend which the students will receive, the extra lectures they will be able to have under Government supervision and the summer training camp they will be required to attend. None of us will know about this plan until some time during the summer, but all colleges were asked to consider it until more definite steps could be taken concerning it."

---

### Cure Flu With Orange Juice.

The dwellers in Barrio Borinquen, an outlying district of Aguadilla, have apparently discovered an effective remedy for influenza. Treating it as a simple grip attack they take orange juice and live on a vegetable diet. As a result, out of 300 cases there has been but one fatality, and that was a case where poverty and abandonment played a principal part.

---

### Physicians of London To Organize Trade Union.

Men and women doctors in London have set about to secure what they deem to be their rights through the organization of a trade union.

The union, which is being formed under the direction of the Medico Political union, is for the purpose of dealing with the government through the latter's proposed ministry of health. It is the purpose of the union to become affiliated with the labor party and the trade union congress.

